# Dependency criterion based brain pathological age estimation of Alzheimer’s disease patients with MR scans

**DOI:** 10.1186/s12938-017-0342-y

**Published:** 2017-04-24

**Authors:** Yongming Li, Yuchuan Liu, Pin Wang, Jie Wang, Sha Xu, Mingguo Qiu, For the Alzheimer’s Disease Neuroimaging Initiative (ADNI)

**Affiliations:** 1 College of Communication Engineering, Chongqing University, Shapingba District, Chongqing, 400044 China; 2 Department of Medical Image, College of Biomedical Engineering, Third Military Medical University, Chongqing, 400038 China; 3 Collaborative Innovation Center for Brain Science, Chongqing University, Chongqing, 400044 China

**Keywords:** Brain age estimation, Brain pathological age, Alzheimer’s disease, Classification, Correlation criterion, Magnetic resonance imaging, Support vector regression

## Abstract

**Objectives:**

Traditional brain age estimation methods are based on the idea that uses the real age as the training label. However, these methods ignore that there is a deviation between the real age and the brain age due to the accelerated brain aging.

**Methods:**

This paper considers this deviation and obtains it by maximizing the correlation between the estimated brain age and the class label rather than by minimizing the difference between the estimated brain age and the real age. Firstly, set the search range of the deviation as the deviation candidates according to the prior knowledge. Secondly, use the support vector regression as the age estimation model to minimize the difference between the estimated age and the real age plus deviation rather than the real age itself. Thirdly, design the fitness function based on the correlation criterion. Fourthly, conduct age estimation on the validation dataset using the trained age estimation model, put the estimated age into the fitness function, and obtain the fitness value of the deviation candidate. Fifthly, repeat the iteration until all the deviation candidates are involved and get the optimal deviation with maximum fitness values. The real age plus the optimal deviation is taken as the brain pathological age.

**Results:**

The experimental results showed that the separability of the samples was apparently improved. For normal control- Alzheimer’s disease (NC-AD), normal control- mild cognition impairment (NC-MCI), and mild cognition impairment—Alzheimer’s disease (MCI-AD), the average improvements were 0.164 (31.66%), 0.1284 (34.29%), and 0.0206 (7.1%), respectively. For NC-MCI-AD, the average improvement was 0.2002 (50.39%). The estimated brain pathological age could be not only more helpful for the classification of AD but also more precisely reflect the accelerated brain aging.

**Conclusion:**

In conclusion, this paper proposes a new kind of brain age—brain pathological age and offers an estimation method for it that can distinguish different states of AD, thereby better reflecting accelerated brain aging. Besides, the brain pathological age is most helpful for feature reduction, thereby simplifying the relevant classification algorithm.

## Background

Alzheimer’s disease (AD) is a common neurodegenerative disease. The key for prevention and treatment is early diagnosis [1]. Magnetic resonance imaging (MRI) is a medical imaging technique used in radiology to visualize the anatomy and the physiological processes of the body in both healthy and disease states. It is noninvasive, nonradioactive, and highly cost-effective, and it can reflect changes in anatomical structures and functions in different biological tissue quantitatively, so it has been applied in the early diagnosis of AD with positive results [2, 3]. Research on AD based on MRI has been conducted according to the visible changes for diagnosis [4–7]. Although research has obtained positive results, the classification accuracy, stability and the number of biomarkers are still not sufficient for clinical applications.

Brain MR Images include some changes invisible to the naked eye, such as $A\beta$ plaque deposition, asymmetry, age, and so on [8–11]. These changes usually represent more essential information about the evolutionary process of AD [12–17]. MRI could be helpful for a deeper understanding of the development process of the disease and for providing better image biomarkers, thereby realizing better classification accuracy with fewer features.

Among these features, brain age is a representative biomarker [16–18]. Pfefferbaum et al. found that the volumes of the major anatomical structures changed as age increased. The anatomical structures include gray matter (GM), white matter (WM), and cerebrospinal fluid (CSF) [19]. It was found that there are complex relationships between changes in the anatomical structures and normal aging [20]. Good et al. found that a linear decrease in GM was predominant in normal aging, as well as a decrease in CSF, according to a cross-sectional Voxel-Based Morphometry (VBM) study [20]. In addition, local areas of GM decrease with age, and cross-sectional morphometric analysis suggested that there are non-linear patterns of neurodegenerative age-related changes in GM volume [21]. Cole et al. found that the brain age can reflect the accelerated atrophy after traumatic brain injury [22]. Rzezak found that the age-related changes in gray matter relate to the education attainment [23]. Duchesne et al. estimated the brain age across the life span using MRI technique [24].

There is a strong relationship between age-related changes in the anatomical structures and the neurodegenerative diseases, such as Alzheimer’s disease (AD), vascular dementia (VD) and schizophrenia [25–27]. Even before the onset of clinical symptoms, some anatomical structures begin to undergo accelerating changes, including volume decreases compared with normal aging. In other words, the age feature has become very important for neurodegenerative diseases, especially AD, so it has received much attention until now [20, 21, 26]. Most of the results have been positive and have shown the feasibility of early diagnosis. In addition, these results have supported the fact that AD is a form of accelerated aging, indicating accelerated brain atrophy [26–29, 36].

Due to the importance of the age feature, some studies of age estimation have been conducted using MRI scans in recent years [30–32]. The research has shown that it is feasible and effective to noninvasively estimate brain age using MRI scans. Some research has further studied how to estimate the brain age on MRI scans in some age-related diseases, including AD [33–40, 45]. The results have shown that it is feasible to estimate age using MRI images. Some of these studies used only NC samples (healthy people) for training age estimation models in order to estimate the distinguishable brain age for the diagnosis of AD. The estimated distinguishable brain age could be determined in different classes of samples [36–39].

Encouraged by the role of the estimated brain age on diagnosis of AD, the researchers further studied the improvements and applications of the brain age estimation method [40–46]. Irimia, Andrei et al. combined the structural and connectome information in sMRI and DTI images for brain age estimation [40]. Kondo et al. parcellated brain tissues into local regions defined by the automated anatomical labeling atlas and extracted the features of the local regions for brain age estimation [41]. Nakano et al. conducted brain age estimation by using Manifold learning, principal component analysis, and multiple regression models [42]. Except from the improved studies above, recently some application studies based on the brain age estimation were conducted [43, 44]. Loewe et al. further combined estimated brain age and APOE status for classification of AD and MCI patients [43]. Luders et al. studied the difference between the estimated brain ages of the long-term meditators and the control subjects [44]. The Katja Franke et al. studied the effect of the APOE Genotype on individual brain age in normal aging, Mild Cognitive impairment, and Alzheimer’s disease. They found that the brain age can be a useful and accurate tool for predicting conversion from MCI to AD even if the information of the patient’s APOE status is missing [45].

It is worth noting that the studies above were based on the same idea to estimate the brain age. The idea was to estimate the brain age by minimizing the difference between the estimate age and the real age. Firstly, a regression model was selected to estimate the age; the input consisted of the MR image features, and the output is the estimated age. Secondly, an error function was designed to train the model such as mean absolute error (MAE) which was the difference between the estimate age and the real age. Thirdly, it found the best estimated age by minimizing the error.

As to the idea, there are some problems to consider. Firstly, because AD is a type of accelerated aging, the deviation between the real age and the brain age changes with different states NC, MCI, and AD. Therefore, it is not suitable to use the real age as the training label. Secondly, the traditional methods aimed to estimate an age close to the real age by minimizing the error function (distance). Because the real age is not suitable for the training label, the minimization is meaningless for classification. Thirdly, some studies have trained regression models with NC samples and tested people with three states (NC MCI AD), indicating that the NC samples contained information about the difference between NC, MCI and AD. However, evidence or proof has not been provided in these papers. The age estimation was based on regression (machine learning method), but the training samples (NC) were quite different from the test samples (NC, MCI, and AD). According to machine learning theory, the training process was not reasonable and reliable.

Because the fact is that the AD process is a form of accelerated aging, the deviation between the real age and estimated brain age should be considered. The training label should not be the real age but the real age plus deviation. Because the deviation is related to the evolutionary process of AD, it can characterize the three states of AD. Therefore, it would be reasonable to determine the suitable deviation by maximizing the classification accuracy of the three states of AD. These deviations could quantitatively and directly estimate the extent of accelerated aging, improve the classification ability of the estimated age and be helpful for the early diagnosis of AD and for understanding neurodegenerative diseases. Because the age deviations are related to the diagnosis of AD, the age plus deviation is called the brain pathological age here.

## Methods

### Subjects/database

In order to validate the algorithm in this paper, the paper selected the publicly accessible ADNI database (http://adni.loni.usc.edu/). The samples were chosen with preprocessing and feature extraction, while in order to emphasize the role of age and to avoid the impact and fluctuation of the multiple features, the samples had only 2 image features and had not been processed with feature selection. The two features of the data set were the volumes of the left and right parts of the hippocampus. The total number of samples in the data set was 1485, consisting of three classes of samples: NC, MCI and AD. The number of NC samples was 540, the number of MCI samples was 534, and the number of AD samples was 411. The age distribution ranges of the three classes of samples were all 65–85 years old. The MRI sequence used is T2 dual echo sequence at 1.5T; the image size is about $256 \times 256 \times 170$ voxels with the voxel size of approximately 1 mm × 1 mm × 1.2 mm. The image scanner was a GE Medical Systems scanner. With the SPM8 package and the VBM8 toolbox, two features are extracted from the MRI images, and the features are the volumes of left and right hippocampus, thereby obtaining the feature data. The feature data is stored with excel format in the ADNI. Since same images with different image processing methods will lead to different feature data, thereby influencing the comparison of different brain age estimation methods. Hence the feature data rather than the relevant images are used for study directly in this manuscript.

To simplify the analysis, the samples were divided into three classes: NC, MCI and AD. Moreover, the numbers of the three classes of samples were the same in order to eliminate the effects of unbalanced samples. The number of AD samples was 411 or less, so the number of different classes of samples was 411. The three classes of samples were within similar age distribution ranges of 65–85 years old. To facilitate description, the data set is called the “hippocampus dataset” in subsequent sections. Relevant, brief information about the hippocampus dataset is shown in Table 1.

**Table 1:** Basic information about the hippocampus dataset

Class	Number	Age range (years)	Mean age (years)	Age standard deviation	Men/women
NC	411	65–85	76.092	4.696	185/226
MCI	411	65–85	75.362	7.635	234/177
AD	411	65–85	75.503	7.245	198/223

### The difference between Path_brainAge_estima and BrainAge_estima

BrainAge_estima means the traditional method for brain age, and the Path_brainAge_estima means the proposed method for brain age in this paper. The fitness function of the training model of the BrainAge_estima was the error of estimated age and real age,and the algorithm estimated the age by minimizing the error. The purpose was to train the age estimation model to approximate the real age. In this paper, the fitness function of the training model of the Path_brainAge_estima was based on the correlation criterion, namely the correlation of the estimated age and the class label. The proposed algorithm estimated the brain age by maximizing this correlation value which indirectly reflects the classification capability. Its purpose was to train the age estimation model to approximate the optimal classification accuracy. Compared with the BrainAge_estima, this Path_brainAge_estima was not only based on the optimization of classification accuracy but also reflected the fact mentioned in the Introduction section. The estimated pathological brain age was more beneficial to improving the classification accuracy for the diagnosis of AD (classification of AD).

The fitness functions of the two types of algorithm are briefly described as follows.$F_{1}$ is the fitness function of the BrainAge_estima, and $F_{2}$ is the fitness function of the Path_brainAge_estima in this paper.

1 $$F_{1} = \arg [min\left( {\left\| {\hat{y} - y} \right\|_{\alpha } } \right)]$$

where $y$ is the real age, and $\hat{y}$ is the age estimated by the regression model.

2 $$F_{2} = \arg [max\left( {corr(\hat{y},y_{label} )} \right)]$$

where $\hat{y}$ is the age estimated by the regression model, and $y_{label}$ is the class label of samples.

### The Path_brainAge_estima

In this paper, we present an idea for automatically estimating the brain pathological ages of subjects with different states of AD using MRI images (scans) for the diagnosis of AD. Firstly, the deviation is considered to characterize accelerated aging directly. Secondly, the training label is real age plus deviation, so the objective of training the age estimation model becomes more reasonable. Thirdly, a fitness function is designed with the correlation criterion so that the deviation can contribute to improving the diagnosis of AD. As we know, the aim of estimating the brain age is to diagnose AD, so the estimation of brain age can be transformed into a maximization problem. Fourthly, the training samples include subjects with the three states of AD, so the whole process of estimating the brain age not only uses information from NC samples but also information from subjects of MCI and AD. The information for training the age estimation model is more abundant and helps to improve the quality of the estimated deviation. The real age plus the estimated deviation is called the brain pathological age. During optimizing the brain age estimation algorithm, different kernel functions and unbalanced/balanced datasets are studied to choose the best brain age estimation model.

To verify the performance of this proposed algorithm, subjects from a public dataset and cross validation (CV) testing methods were used. The dataset came from a popular public dataset of AD-related research: ADNI (Alzheimer’s Disease Neuroimaging Initiative, ADNI). In total, data from more than 1200 subjects were included. Two-class classification experiments (NC-MCI, NC-AD, and MCI-AD) were performed. In addition, the three-class classification problem was also considered. Except for the classification problems, the deviation was discussed so that the estimated age could show strong separability capability for different states of AD. Each experiment was repeated several times to demonstrate the stability, statistical characteristics and significance level of the estimated age. Because the traditional age estimation methods for diagnosis of AD are based on the same idea, the comparison was not based on one concrete algorithm but based on the idea. Therefore, in the experimental part, only one representative algorithm in [36] was selected and compared.

To facilitate description, the proposed age estimation idea (algorithm) and estimated age are called Path_brainAge_estima and brain pathological age respectively. The traditional brain age idea (algorithm) and the estimated age are called BrainAge_estima and traditional brain age respectively.

The proposed algorithm in this paper (Path_brainAge_estima) was mainly based on a hybrid integrated age deviation selection model, by searching the age deviation to maximize the fitness function (2) and to obtain the brain pathological age. It mainly included the following parts: (1) the regression model—support vector regression (SVR); and (2) a fitness function (evaluation criteria): correlation criterion. Because the brain pathological age and real age have a deviation and it changes with the state of AD, the deviation is a variable.

For class 1, the deviation is set to $w$, which ranges from $w_{\text{min} }$ to $w_{\text{max} }$; the deviation for class 2 is set to $q$, which ranges from $q_{\text{min} }$ to $q_{\text{max} }$; the deviation for class 3 is set to $r$, which ranges from $r_{\text{min} }$ to $r_{\text{max} }$,…; the deviation of class n is set to be $s$, which ranges from $s_{\text{min} }$ to $s_{\text{max} }$. Assuming that the real age of the $i1th$ samples in class 1 is $A_{ge\_class1\_i1}$, the $i2th$ sample in class 2 is $A_{ge\_class2\_i2}$, the $i3th$ sample in class 3 is $A_{ge\_class3\_i3},\ldots,$ the $inth$ sample in class n is $A_{{ge\_class{\text{n}}\_in}}$, and the training label of the SVR is not $A_{ge\_class1\_i1}$, $A_{ge\_class2\_i2}$, $A_{ge\_class3\_i3} \ldots,$ $A_{{ge\_class{\text{n}}\_in}}$ but $A_{ge\_class1\_i1} + w$, $A_{ge\_class2\_i2} + q$, $A_{ge\_class3\_i3} + r, \ldots$, $A_{{ge\_class{\text{n}}\_in}} + s$, respectively.

Firstly, the samples are divided into a training set, a validation set and a test set randomly. Secondly, the SVR model is trained using the training set based on the current combination of deviations: $w$,’$q$, $r$,…, $s$. Then, the input validation set is inserted into the SVR model to obtain the estimated ages to calculate the fitness value based on the fitness function.

The deviations $w$, $q$, $r$,…, $s$ are within $\left[ {w_{\text{min} } ,w_{\text{max} } } \right]$, $\left[ {q_{\text{min} }, \, q_{\text{max} } } \right]$, $\left[ {r_{\text{min} }, \, r_{\text{max} } } \right]$,…,$\left[ {s_{\text{min} }, \, s_{\text{max} } } \right]$, respectively, and all the candidate deviations belong to the set of $F_{2} \{ \} \left| {_{w,q,r, \ldots,s} } \right.$. The $F_{2} \{ \} \left| {_{w,q,r, \ldots, s} } \right.$ is defined as follows: $\left\{ {F_{2} \in A_{{F_{2} }} \left| {A_{{F_{2} }} :F_{2} } \right|_{w,q,r,\ldots,s} ,w = w_{\text{min} } :w_{\text{max} } ,q = q_{\text{min} } :q_{text{max} }, r = r_{text{min} } :r_{\text{max} }, \ldots,s = s_{\text{min} } :s_{\text{max} } } \right\}$.In the set, the maximum fitness value $F_{2\_\text{max} }$ is obtained, and the corresponding optimal deviations are $w_{ma}$, $q_{ma}$, $r_{ma}$,…, $s_{ma}$. They are calculated by the following formula:

$$\left[ {w_{ma} ,q_{ma} ,r_{ma} ,\ldots,s_{ma} } \right] = \arg \left\{ {F_{2\_\text{max} } (w_{\text{ma}} ,q_{\text{ma}} ,r_{ma} ,\ldots,s_{ma} )} \right\}$$

**Fig. 1:**
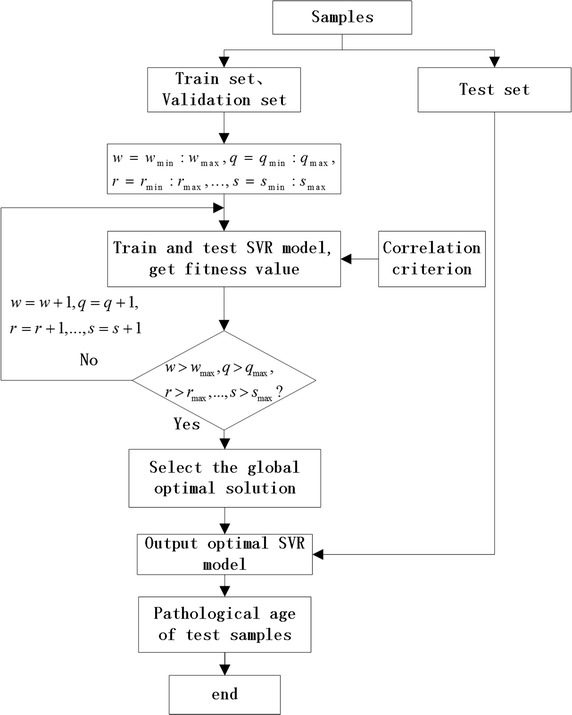
Flowchart of Path_brainAge_estima

The main process of the Path_brainAge_estima is described by the following flowchart in Fig. 1.

As seen from the flowchart, this Path_brainAge_estima uses the SVR model to estimate brain pathological age and introduces the separability distance criterion to design the fitness function. The algorithm fully considers that there are different deviations between the real age and brain age in different states of AD. The pseudo-code of this algorithm is shown as follows. The process of this algorithm is to calculate the fitness value based on a combination of deviations $\left( {w,q,r,\ldots,s} \right)$. Therefore, it is necessary to repeat the following circles: $\left[ {w_{\text{min} } ,w_{\text{max} } } \right]$, $\left[ {q_{\text{min},} \, q_{\text{max} } } \right]$, $\left[ {r_{\text{min} }, \, r_{\text{max} } } \right]$,…, $\left[ {s_{\text{min} }, \, s_{\text{max} } } \right]$, while computing and storing all possible combinations of $w,q,r,\ldots,s$, corresponding to the fitness values and the corresponding trained SVR models.

The pseudo code is described as follows: 

#### Support vector regression (SVR)

Support Vector Machine (SVM) is a highly efficient type of machine learning algorithm described by Vapnik. SVR is the regression variation of SVM with outstanding nonlinear mapping performance [47]. The purpose of SVR is to determine the plane that can accurately predict the distribution of the data. If the problem is linear, the equation for the hyperplane is provided by expression (3):

3 $$f\left( x \right) = \frac{1}{n}\sum\limits_{i = 1}^{n} {\lambda_{i}^{*} } y_{i} \left\langle {x} \mathrel{\left | {\vphantom {x {x_{i} }}} \right. \kern-0pt} {{x_{i} }} \right\rangle + b^{*}$$

where $\lambda^{*}$ and $b^{*}$ are Lagrange multipliers.

If the problem is nonlinear, there are two methods to obtain a linear case. The first idea is that the data are projected in a space with a greater dimension; the other idea is the introduction of a kernel function. The SVR is used here for its robustness against noise and the possibility of processing data that are nonlinear. In this paper, the outputs of SVR are the estimated ages.

#### Fitness function based on dependency criterion

Correlation quantifies the relationship between features in order to identify feature candidates that may be the best to achieve desired effects [48, 49]. Linear correlation methods are robust and computationally efficient, but only detect the linear correlations. Nonlinear correlation methods can detect nonlinear correlations, but require careful parameterization. Nonlinear correlations can also be quantified by regression validation errors. Correlations do not imply causality, so correlation analysis may reveal false correlations. If the underlying features are known, the spurious correlation can be handled in a partially correlated way. Suppose that the covariance matrix **Ϲ** of a data set $X \subset R^{F}$,where each matrix element $c_{ij}$ denotes the covariance between the features $x^{(i)}$ and $x^{(j)}$, i, j = 1,…, p.

4 $$C_{ij} = \frac{1}{n - 1}\sum\limits_{k - 1}^{n} {\left( {x_{k}^{(i)} - \overline{x}^{(i)} } \right)\left( {x_{k}^{(j)} - \overline{x}^{(j)} } \right)} \,$$

If $c_{ij}$ is positive, then there is a strong positive dependency between $x^{(i)}$ and $x^{(j)}$, i.e. high values of $x^{(i)}$ coincide with high values of $x^{(j)}$, and low values of $x^{(i)}$ coincide with low values of $x^{(j)}$. If $c_{ij}$ is negative, then there is a strong negative dependency, i.e. high values of $x^{(i)}$ coincide with low values of $x^{(j)}$ and vice versa. If $c_{ij}$ is close to zero, then there is a weak dependency between $x^{(i)}$ and $x^{(j)}$. If a feature is multiplied by a constant factor α, then the covariance between this feature and any other feature will also increase by a factor α, although we do not expect this feature to make more useful contributions to data analysis. The correlation coefficient compensates the effect of constant scaling by dividing the covariance by the product of the standard deviations of both features.

5 $$\text{s}_{{\text{ij}}} = \frac{{\text{c}_{{\text{ij}}} }}{{\text{s}^{{\text{(i)}}} \text{s}^{{\text{(j)}}} }}$$

6 $${\text{s}}_{{{\text{ij}}}} = \frac{{\sum\nolimits_{{{\text{k}} = {\text{1}}}}^{{\text{n}}} {\left( {{\text{x}}_{{\text{k}}}^{{{\text{(i)}}}} - \overline{{\text{x}}} ^{{{\text{(i)}}}} } \right)\left( {{\text{x}}_{{\text{k}}}^{{{\text{(j)}}}} - \overline{{\text{x}}} ^{{{\text{(j)}}}} } \right)} }}{{\sqrt {\left( {\sum\nolimits_{{{\text{k}} = {\text{1}}}}^{{\text{n}}} {{\text{(x}}_{{\text{k}}}^{{{\text{(i)}}}} - \overline{{\text{x}}} ^{{{\text{(i)}}}} {\text{)}}^{{\text{2}}} } } \right)\left( {\sum\nolimits_{{{\text{k}} = {\text{1}}}}^{{\text{n}}} {{\text{(x}}_{{\text{k}}}^{{{\text{(j)}}}} - \overline{{\text{x}}} ^{{{\text{(j)}}}} {\text{)}}^{{\text{2}}} } } \right)} }}$$

7 $${\text{s}}_{{{\text{ij}}}} = \frac{{\sum\nolimits_{{{\text{k}} = {\text{1}}}}^{{\text{n}}} {{\text{x}}_{{\text{k}}}^{{{\text{(i)}}}} {\text{x}}_{{\text{k}}}^{{{\text{(j)}}}} } - {\text{n}}\overline{{\text{x}}} ^{{{\text{(i)}}}} \overline{{\text{x}}} ^{{{\text{(j)}}}} }}{{\sqrt {\left( {\sum\nolimits_{{{\text{k}} = {\text{1}}}}^{{\text{n}}} {{\text{(x}}_{{\text{k}}}^{{{\text{(i)}}}} {\text{)}}^{{\text{2}}} - {\text{n}}} {\text{(}}\overline{{\text{x}}} ^{{{\text{(i)}}}} {\text{)}}^{{\text{2}}} } \right)\left( {\sum\nolimits_{{{\text{k}} = {\text{1}}}}^{{\text{n}}} {{\text{(x}}_{{\text{k}}}^{{{\text{(j)}}}} {\text{)}}^{{\text{2}}} - {\text{n}}} {\text{(}}\overline{{\text{x}}} ^{{{\text{(j)}}}} {\text{)}}^{{\text{2}}} } \right)} }}$$

The standard deviations are the square roots of the variances, i.e. the square roots of the diagonal elements of the covariance matrix, $s^{(i)} = \sqrt {c_{ii} }$, so the correlation matrix can be directly computed from the covariance matrix.

8 $$s_{ij} = \frac{{c_{ij} }}{{\sqrt {c_{ii} c_{jj} } }}$$

where $s_{ij}$ ∈ [−1,1]. If $s_{ij}$ ≈ 1 then there is a strong positive correlation between $x^{(i)}$ and $x^{{({\text{j}})}}$. If $s_{ij}$ ≈ −1, then there is a strong negative correlation. If $s_{ij}$ ≈ 0 then $x^{(i)}$ and $x^{{({\text{j}})}}$ are (almost) independent, so correlation can be interpreted as the opposite of independence. Notice that for μ–σ—standardized data, covariance and correlation are equal.

This paper designed correlation criteria as fitness functions. Select the correlation between the predicted age of the validation set and the category label as the fitness value. The fitness function of the expression is:

9 $$\lambda = corr(\hat{y},y_{label} ) = s_{i,j}$$

where λ is fitness value, $\hat{y}$ is the estimated brain age and $y_{label}$ is the categories of samples.

### Experimental conditions

In order to demonstrate the advantages of this proposed brain pathological age estimation algorithm, two fitness functions were used: $\lambda_{1}$ and $\lambda_{2}$. A two-class experiment and three-class experiment were conducted, which were NC-AD, NC-MCI, MCI-AD and NC-MCI-AD. The samples were randomly divided into a training set, a validation set and a test set 100 times, yielding 100 groups of samples.

In this paper, the experimental operating system platform was the Windows, version 7, 64-bit operating system, and the memory size was 128 GB. The algorithm was implemented in MATLAB, version 2014a. Because the Path_brainAge_estima is different from the traditional age estimation idea (BrainAge_estima) rather than a concrete algorithm, only one representative algorithm based on the traditional idea [36] was selected, realized and compared with the Path_brainAge_estima (the proposed algorithm). The kernel functions of SVR include linear kernel, Polynomial kernel and Gaussian kernel; the parameters are set with default values.

## Results

### Estimation of brain pathological age

#### Study of kernel function of SVR

For the two classes of samples, the range of age deviation is set according to prior knowledge. The deviation is usually within 10 years or so. Therefore, $\left[ {w_{\text{min} } ,w_{\text{max} } } \right] = [ -10,10]$, $\left[ {q_{\text{min} } ,q_{\text{max} } } \right] = [ -10,10]$. In order to compare the results of different kernel functions, the experiment was repeated 10 times with different kernel functions respectively for NC-AD, and kernels’ parameters are the default. The average results about age detection $\left( {w,q} \right)$ are shown in Table 2.

**Table 2:** Results about age detection $w,q$ with different kernel functions for NC-AD

Experiment methods	Polynomial kernel	Gaussian kernel	Linear kernel
	Mean	Mean	Mean
Without age detection	(0, 0)	(0, 0)	(0, 0)
Path_brainAge_estima (w, q)	(−2.5, 4.1)	(−5.3, 3)	(−5.1, 7)
Significant difference (P value)	(0.255, 0.0285)	(0.0262, 0.0714)	(0.0211, 0.0005)

From Table 2, for NC-AD, it can be seen the average values of $w$ were always less than $q$, but the difference between the $w$ and $q$ obtained by the linear kernel function was the largest in the three kernel functions. The average values of $w$ was −5.1 and the average values of $q$ was 7 with linear kernel function, respectively. $w$ was usually less than zero, and $q$ was normally greater than zero. So the pathological age obtained by the linear kernel function could distinguish between healthy people and AD patients best.

The age estimation was based on the training samples and validation samples. The training samples were used for training the age estimation model. The validation samples were used to calculate the fitness value of the deviation candidate and to determine the optimal brain pathological age and the optimal age estimation model. To further verify the performance of the optimal age estimation model, it is necessary to apply the model to the test samples.

In this section, experiments about NC-AD with different kernel functions are conducted. The correlation coefficient is used as dependency criterion here. As discussed above, the values based on the dependency criterion can detect the correlation of the test samples better, which in turn can improve classification accuracy indirectly. If the correlation value is large, then classification accuracy is high accordingly. The mean and standard deviation of the correlation values obtained by different kernel functions are shown in Table 3.

**Table 3:** Comparison of brain age with different kernel functions for NC-AD (correlation coefficient)

	Polynomial kernel		Gaussian kernel		Linear kernel	
	Mean	Std	Mean	Std	Mean	Std
Without age estimation (w = 0, q = 0)	0.059	0.0363	0.0592	0.0363	0.0592	0.0363
BrainAge_estima	0.558	0.0179	0.6746	0.0230	0.518	0.2354
Path_brainAge_estima	0.625	0.0181	0.6785	0.0243	0.682	0.0235

From Table 3, for different kernel functions, the Path_brainAge_estima showed better correlation than the case **‘**without age estimation**’**, which indicate that real age alone was not sufficient, In addition, our algorithm had better correlation (see the boldface type) than the current popular idea (BrainAge_estima). For normal control- Alzheimer’s disease (NC-AD), normal control- mild cognition impairment (NC-MCI), and mild cognition impairment—Alzheimer’s disease (MCI-AD), the average improvements were 0.164 (31.66%), 0.1284 (34.29%), and 0.0206 (7.1%), respectively.

The improvements are apparent, especially with linear kernel function. It can be found that the mean of fitness value with linear kernel was the largest for the three kernel functions. It showed that the brain pathological age from our algorithm with linear kernel function was more helpful for the classification of AD. Therefore, it is applied for the subsequent experiments.

#### Estimation of brain pathological age

For the two classes of samples, experiments about NC-MCI and MCI-AD were also conducted for 10 times respectively; the range of the deviation was set as the same as NC-AD; the average results about age detection $\left( {w,q} \right)$ are shown in Table 4. Considering the time cost, for three classes of samples, the range of age deviation was set as follows: $\left[ {w_{{_{\text{min} } }} ,w_{\text{max} } } \right] = [ - 8,8]$, $\left[ {q_{\text{min} } ,q_{\text{max} } } \right] = \left[ { - 8,8} \right]$, $\left[ {r_{\text{min} } ,r_{\text{max} } } \right] = \left[ { - 8,8} \right]$. The same experiment was repeated 10 times. The average results for the age estimation of $\left( {w,q,r} \right)$ are shown in Table 5.

**Table 4:** Results for age detection $w,q$

Experiment methods	NC-AD ($w,q$)	NC-MCI ($w,q$)	MCI-AD ($w,q$)
	Mean	Mean	Mean
Without age estimation	(0, 0)	(0, 0)	(0, 0)
Path_brainAge_estima	( −5.1, 7)	( −3.9, 0.7)	(−2.2, 2.4)
Significant difference (P value)	(0.0211, <0.001)	(0.0797, 0.7797)	(0.2645, 0.21)

**Table 5:** Results for age detection $w,q,r$

Methods	NC-MCI-AD ($w,q,r$) mean
Without age estimation	(0, 0, 0)
Path_brainAge_estima	(−4, − 0.7, 2.7)
Significant difference (P value)	(0.0119, 0.6188, 0.1535)

**Fig. 2:**
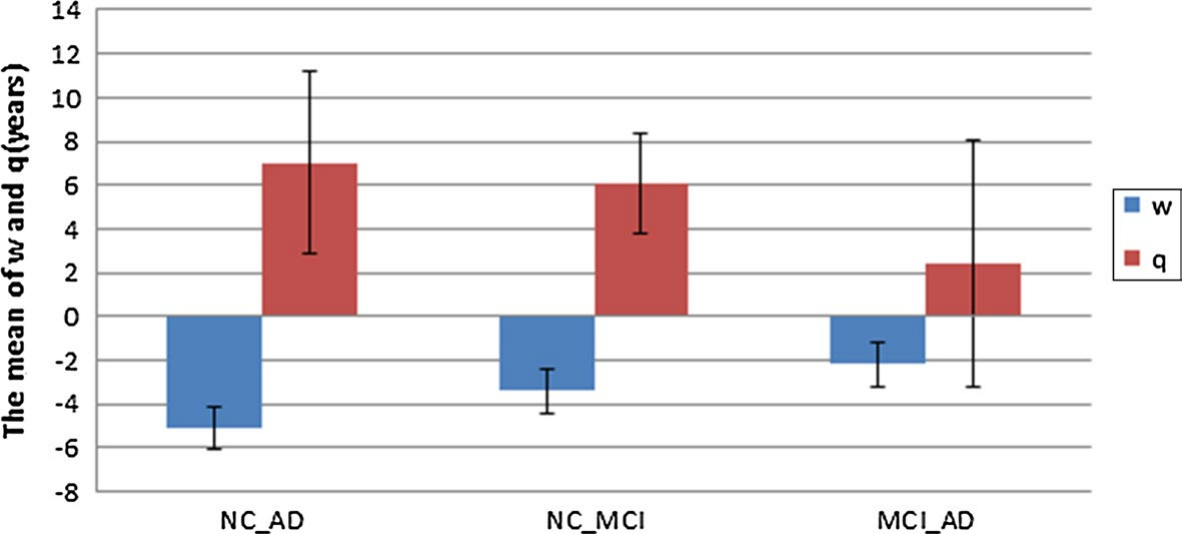
Averages values of $w,q$ in the two-class problem. $w$ is the deviation between the real age and the brain pathological age of the class 1 samples. $q$ is the deviation between the real age and the brain pathological age of the class 2 samples

From Table 4, for NC-AD, it can be seen the average values of $w$ was −5.1 and the average values of $q$ was 7, respectively. $w$ was always less than $q$. In addition, it was also found that $w$ was usually less than zero, and $q$ was normally greater than zero. There was a difference between healthy people’s pathological age and AD patients’ pathological age. In other words, the pathological age could distinguish between healthy people and AD patients while the real age could not. In order to show the significant difference between the pathological age and the real age, p-values are computed. According to the p-values, two of the estimated pathological ages were significantly different from the real age (p < 0.05) significantly. The case was similar with NC-MCI and MCI-AD, and $w$ was always less than $q$ (see Fig. 2).

**Fig. 3:**
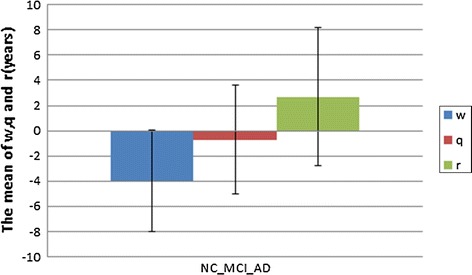
Averages of $w,q$ and $r$ in the three-class problem. $w$ is the deviation between the real age and the brain pathological age of the class 1 samples. $q$ is the deviation between the real age and the brain pathological age of the class 2 samples. $r$ is the deviation between the real age and the brain pathological age of the class 3 samples

From Table 5, for the three classes of samples (NC-MCI-AD), it could be seen that the average value of $w$ was −4, $q$ was −0.7, and $r$ was 2.7, and they meet the inequality constraints $w < q < r$. The results showed that the deviation for healthy people (NC) was usually lower than that for the MCI subject, and the latter is lower than that for AD patients. In other words, the deviation between the pathological age and the real age could distinguish NC, MCI and AD, while the single real age could not. Please see Fig. 3 for more information.

### Verification of effectiveness of the estimated brain pathological age

In this section, the two-class and three-class problems are carried out. They are NC-MCI, MCI-AD, and NC-MCI-AD. As the same as Table 3, the mean and standard deviation of the correlation values are shown in Tables 6 and 7 for the two-class problem and three-class problem.

**Table 6:** Comparison of brain age for two types of sample (correlation coefficient)

	NC-AD		NC-MCI		MCI-AD	
	Mean	Std	Mean	Std	Mean	Std
Without age estimation (w = 0, q = 0)	0.059	0.0363	0.071	0.0412	0.035	0.0257
BrainAge_estima	0.518	0.2354	0.3744	0.1909	0.29	0.0681
Path_brainAge_estima	0.682	0.0235	0.5028	0.0403	0.3106	0.0569

**Table 7:** Comparison of brain age for the three classes of sample (correlation coefficient)

NC-MCI-AD		
Different methods	Fitness value	
	Mean	Std
Without age estimation (w = 0, q = 0, r = 0)	0.0449	0.0277
BrainAge_estima	0.3973	0.1822
Path_brainAge_estima	0.5975	0.0232

From Table 6 above, for the cases of NC-AD, NC-MCI and MCI-AD, the Path_brainAge_estima showed better correlation than the case **‘**without age estimation**’**, which indicate that real age alone was not sufficient, so it was necessary to estimate the brain age. In addition, our algorithm had better correlation value (see the boldface type) than the other algorithms. It showed that the pathological age from our algorithm was more helpful for the classification of AD. According to the case **‘**without age estimation**’**, Path_brainAge_estima and BrainAge_estima showed apparent improvements, but these improvements were different with the two-class problems. For NC-AD, the improvement was most apparent, possibly because NC is quite different from AD. According to the standard deviation, our algorithm was better than the BrainAge_estima, indicating that our algorithm was more stable than the traditional age estimation algorithm. For MCI-AD, all the correlation values are lower than 0.5, it means that the correlation is not strong enough. The possible first reason is that the difference between MCI and AD are small and they are difficult to be separated. The possible second reason is that the step size for the brain pathological age estimation is not small enough and the search range of the brain age deviationmay not be appropriate.

The case is similar to that in Table 7. From the Tables above, for the cases of NC-MCI-AD, the BrainAge_estima had better correlation than the case **‘**without age estimation**’**, indicating that real age alone was not sufficient and that it was necessary to estimate the brain age. In addition, our algorithm had better correlation (see the boldface type) than the traditional age estimation algorithm, demonstrating that the brain pathological age from our algorithm was more helpful to the classification of AD. According to the case ‘without age estimation’, BrainAge_estima and Path_brainAge_estima had apparent improvements. The difference between healthy people and AD patients was amplified as much as possible. Nevertheless, our algorithm still had the best correlation. According to the standard deviation, our algorithm was better than BrainAge_estima, indicating that our algorithm was more stable than the traditional age estimation algorithm.

**Fig. 4:**
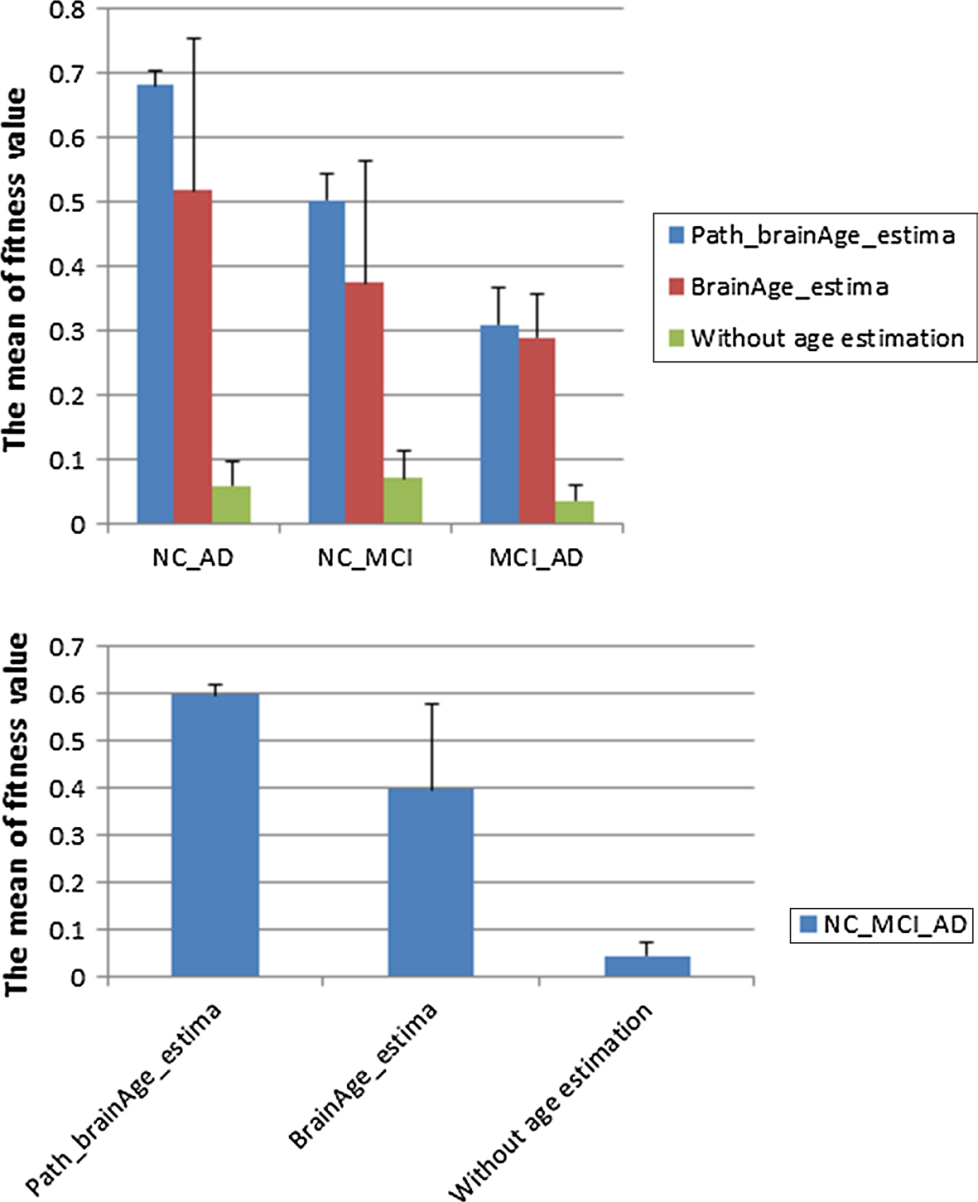
Correlation of test samples based on estimated brain age by different methods. (1) two class; (2) three class

Figure 4 is a graphical representation of Tables 6 and 7, showing that the correlation value with these algorithms had a trend of gradual increase.

As the data analysis above, the brain pathological age has highest correlation values with the class label, thereby having best classification capability. In other words, the brain pathological age has highest dependency with the class label. According to the principle of feature optimization, a good feature subset has two characteristics: high classification capability and small feature size. The high dependency with the class label can support the high classification capability indirectly. In this section, let us study the dependency of the brain age with the MR features. The high dependency can support the high redundancy, thereby being helpful for reducing the feature size. Table 8 shows the dependency of the brain age with the class label and the MR features. ‘CwC’ means correlation of age with class label; ‘ACwF’ means average correlation of age with MR features; ‘CwF1’ means the correlation of age with 1st feature; ‘CwF2’ means the correlation of age with 2nd feature. Each data is with format of mean and stand deviation of the correlation value.

**Table 8:** Correaltion of brain age with class label and MR features

Correlation	Without age estimation	BrainAge_estima	Path_brainAge_estima
NC_AD			
CwC	0.059, 0.0363	0.518, 0.2354	0.682, 0.0235
ACwF	0.167, 0.0460	0.735, 0.3411	0.965, 0.0331
CwF1	0.166, 0.0456	0.736, 0.3398	0.994, 0.0053
CwF2	0.169, 0.0487	0.735, 0.3609	0.936, 0.0195
NC_MCI			
CwC	0.071, 0.0412	0.3744, 0.1909	0.5028, 0.0403
ACwF	0.109, 0.0398	0.777, 0.3007	0.959, 0.0322
CwF1	0.108, 0.0378	0.766, 0.3046	0.986, 0.0121
CwF2	0.111, 0.0436	0.788, 0.3128	0.932, 0.0203
MCI_AD			
CwC	0.035, 0.0257	0.29, 0.0681	0.311, 0.0569
ACwF	0.186, 0.0338	0.854, 0.1970	0.929, 0.0799
CwF1	0.186, 0.0280	0.828, 0.2483	0.981, 0.0175
CwF2	0.186, 0.0404	0.879, 0.1372	0.877, 0.0844
NC_MCI_AD			
CwC	0.045, 0.0277	0.397, 0.1822	0.598, 0.0232
ACwF	0.141, 0.0284	0.691, 0.2917	0.961, 0.0342
CwF1	0.139, 0.0213	0.615, 0.3093	0.992, 0.0048
CwF2	0.143, 0.0352	0.766, 0.2670	0.929, 0.0166

Seen from Table 8, the brain age is helpful for improving the dependency with the class label. The brain pathological age obtains highest correlation (dependency) with the class label. It means that the brain pathological age is most helpful for classification of AD. For example, for CwC of NC_MCI_AD, the correlation of the real age with the class label is 0.045, the correlation of the traditional brain age with the class label is 0.397, and the correlation of the brain pathological age with the class label is 0.598. The case is similar as NC_AD, NC_MCI and MCI_AD. More important, the correlation of the brain pathological age with the MR features (redundancy) is highest. It means the brain pathological age is most helpful for feature reduction, thereby reducing the complexity of the classification model. For example, for ACwF of NC_MCI_AD, the correlation of the real age with the MR features is 0.141, the correlation of the traditional brain age with the MR features is 0.691, and the correlation of the brain pathological age with the MR features is 0.961. The case is similar as NC_AD, NC_MCI and MCI_AD.

## Discussion

The estimated brain age, based on MRI images using different methods, can distinguish the different states of AD, and it is helpful for improving classification accuracy. Some methods use all classes of samples for training, while others use only normal people (NC) for training, but all of them are based on the same idea. The idea is to estimate the age by minimizing the distance between the estimated age and the real age. This idea is not in accordance with the fact that AD process is a form of accelerated aging.

This paper solved this problem based on brain pathological age by maximizing the classification accuracy of AD. Firstly, the samples are divided into three sets: training set, validation set and test set. Secondly, age deviation is introduced. Thirdly, the dependency criterion of correlation is used as fitness function. Fourthly, based on the age deviation candidate and the training set, the SVR is trained; the corresponding fitness value is obtained based on the validation set. Fifthly, the age deviation is optimized by maximizing the fitness value and the age deviation candidate with best fitness value is the optimal age deviation. The real age plus the optimal age deviation is called the brain pathological age.

The popular regression method SVR is used as age estimation model. Several kernel functions and dependency criterion are compared in the case of NC_AD. Based on the experimental results, we can find that the age deviation of NC is lower than that of AD. The results demonstrate that the proposed idea works better. The results quantitatively prove the fact that the AD process is a form of accelerated aging. The difference between the age deviation of NC and AD is largest in the case of linear kernel function. The results mean that the linear kernel function is most helpful for maximizing the classification accuracy of NC_AD and is used for subsequent age estimation. The possible reason why the linear kernel function is best is that the kernel function is most suitable for the data. According to correlation values of different kernel functions, the brain pathological age by the proposed algorithm is best. The results show that the proposed age estimation idea is best. Based on the SVR with linear kernel function and the dependency criterion, the proposed age estimation algorithm is applied for cases of NC-AD, NC-MCI, MCI-AD, and NC-MCI-AD. According to the estimated brain age deviations, the accelerating aging is quantitatively calculated. The age deviation of NC is lower that of MCI, the latter is lower than that of AD. In order to show the advantage of the proposed algorithm, the correlation values are calculated in terms of different age types. The correlation value of brain age by existing age estimation method is better than that by real age; the results mean that the brain age estimation is very necessary. The correlation value of brain age by the proposed age estimation algorithm is better than that by existing age estimation method; the results mean that the propose age estimation algorithm is better than the existing brain age estimation method in terms of classification of AD. The reason is in that the age deviations by the proposed brain age estimation algorithm are in accordance with the fact that the AD process is a form of accelerated aging.

Based on the estimated brain ages, the differences between the samples belonging to different classes are calculated. According to the results, the differences by the proposed algorithm not only vary monotonously but also can distinguish the different states of AD. The bar graphs also support this point. The results once again show that the brain pathological age can quantitatively measure the extent of the accelerated aging in the AD process.

The correlation of the brain pathological age and the traditional brain age with the class label and the MR features are studied respectively. The experimental results show that the brain pathological age is most helpful for classification of AD and feature reduction.

At present,all the existing brain age estimation methods are based on same brain age idea which is to minimize the error between the predicted age and the actual age,while is inconsistent with the process of accelerating brain age of AD. In this paper, a new brain age estimation idea (brain pathological age estimation) is proposed and it is quite different from the existing brain age idea. According to the experimental results, for same public feature data, the brain pathological age has higher classification accuracy and can be better helpful for reducing feature size than the existing brain age. The most advantage of the proposed algorithm is that it can improve the accuracy of classification and effectively reduce the feature size. The most limitation of it is that when the number of features is too large and the step size of search brain age deviation is very small, the time cost of the brain pathological age estimation will become high. The potential significance of the algorithm is that this paper proposed a new brain pathological age estimation idea rather than a concrete method, thereby obtaining a new and better brain age type (biomarker). Since there is a new idea, it will lead to many different new concrete methods by introducing different algorithms such as different regression models, optimization algorithms, classification criteria, and so on.

## Highlights

This paper proposed a new kind of brain age-brain pathological age and realized a concrete method for estimating it which is helpful for diagnosis of AD. The main contributions of this paper can be described as follows.

1. The current age estimation methods for the diagnosis of AD are based on the same idea. This paper proposed a new idea to replace it rather than proposed a new concrete method.
2. This proposed idea considers the deviation directly so that it can help to distinguish the different states of AD, thereby estimating the extent of accelerated aging. The age estimation was conducted by maximizing classification accuracy rather than by minimizing the distance between the estimated age and the real age, thus make the estimation helpful for the diagnosis of AD.
3. This idea uses the real age plus deviation as the training label rather than the real age, thereby making the training process more reasonable for the classification of AD.
4. Two states and three states of AD were involved at the same time for brain age estimation in this paper.
5. Dependency criterion of correlation was used for algorithm design and for the verification of the quality of the estimated age. The criterion is a kind of measurement index of classification capability. It has low computational complexity and good generalization capability, so that the brain pathological age can be widely applied in the different individuals from different areas.
6. The brain pathological age is most helpful for feature reduction, thereby reducing the complexity of the classification model.

## Conclusions

Real age has been proven to be related to the classification of AD, but it has poor and unsatisfactory classification capability. From brain MR images, the existing age estimation methods can offer an estimated brain age for classification of AD. But the age estimation methods are based on the same idea, which is to estimate the age by minimizing the distance between the estimated age and the real age. The idea is not in accordance with the AD process. Based on the limitations, this paper proposed a new brain age estimation idea-brain pathological age estimation idea. The experimental results showed that the estimated brain pathological age could reflect the differences between the real age and the brain pathological age at a significant level. The difference could distinguish the different states of AD and was more helpful for the classification of AD, reflecting the extent of accelerated aging better than the traditional brain age estimation idea. Besides, the brain pathological age is most helpful for feature reduction for subsequent classification model.

## Abbreviations

SVR: support vector regression
NC: normal control
MCI: mild cognition impairment
AD: Alzheimer’s disease
MRI: magnetic resonance imaging
GM: gray matter
WM: white matter
CSF: cerebrospinal fluid
VBM: Voxel-Based Morphometry
VD: vascular dementia
DTI: diffusion tensor imaging
MAE: mean absolute error
SVM: Support Vector Machine
